# Bilateral epididymal cyst with spontaneous resolution

**DOI:** 10.1002/ccr3.3199

**Published:** 2020-08-22

**Authors:** Alain Mwamba Mukendi

**Affiliations:** ^1^ Department of Urology Chris Hani Baragwanath Academic Hospital University of the Witwatersrand Johannesburg South Africa

**Keywords:** epididymal cysts, epididymis, scrotal swelling, spontaneous resolution

## Abstract

Bilateral epididymal cysts are infrequent particularly in children. They commonly arise from the epididymal head. Cysts arising from the epididymal tails are very rare. Spontaneous resolution may occur with no need for surgical excision of cysts and can take up to 50 months.

## Graphical abstract

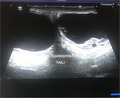

## 1 INTRODUCTION

Bilateral epididymal cysts are infrequent particularly in children. They commonly arise from the epididymal head. Cysts arising from the epididymal tails are very rare. Only few reports are found in the literature. We, herein, report a case of bilateral epididymal tail cysts in a 16‐year‐old male resolved spontaneously.

Epididymal cysts are collection of fluid in a single sac (unilocular) or more than one (multilocular) as a result of dilatation of efferent epididymal tubules due to tubular obstruction.^1^, ^2^ They are benign in nature and mostly unilateral but rarely can be bilateral. Epididymal cysts and spermatoceles on several occasions have been used reciprocally in the literature to describe the same entity.^2^ They can occur at any age, and some authors found that there may be an increase in the proportion of epididymal cysts with age; previous published case reports revealed the condition to be present in about 5% of pediatric patients undergoing scrotal ultrasonography in general.^3^

## 2 CASE REPORT

**FIGURE 1:**
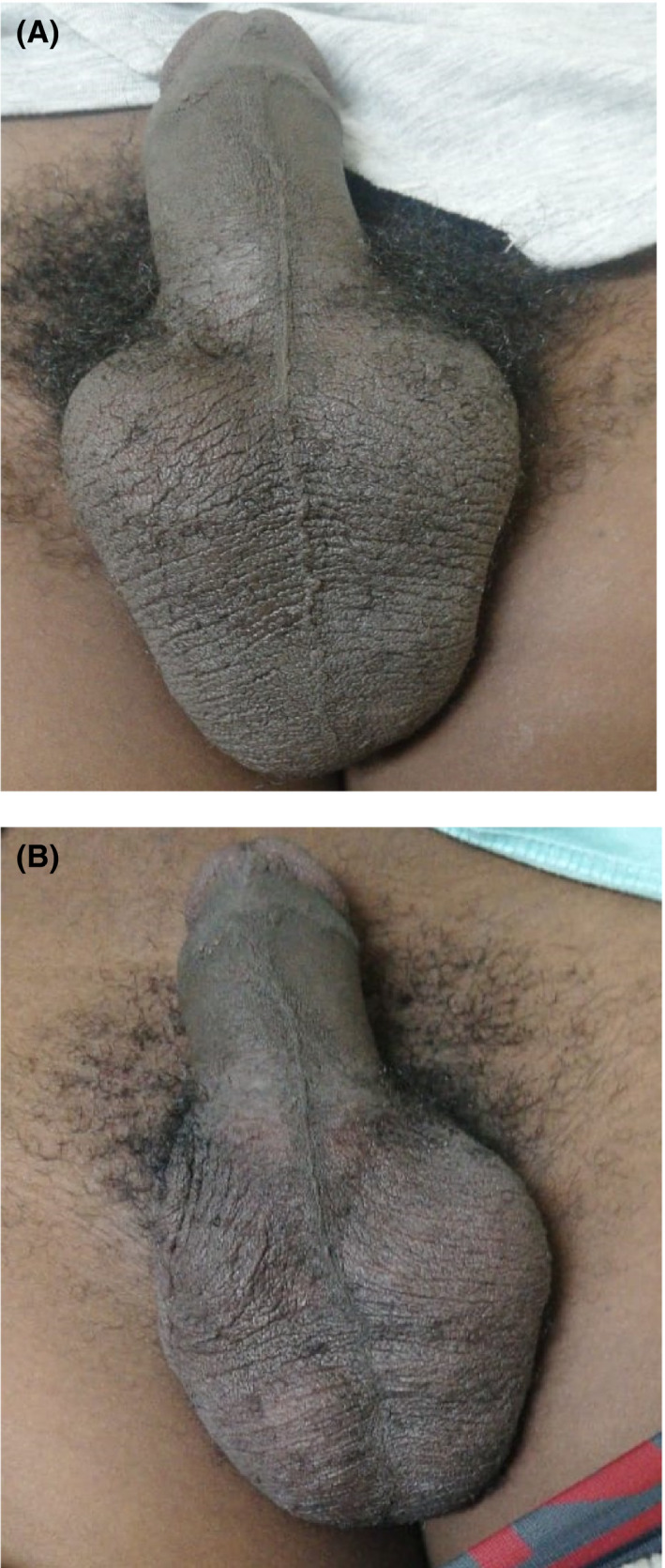
A, Showing bilateral scrotal lumps (epididymal cysts). B, Showing resolution of right‐sided epididymal cyst

**FIGURE 2:**
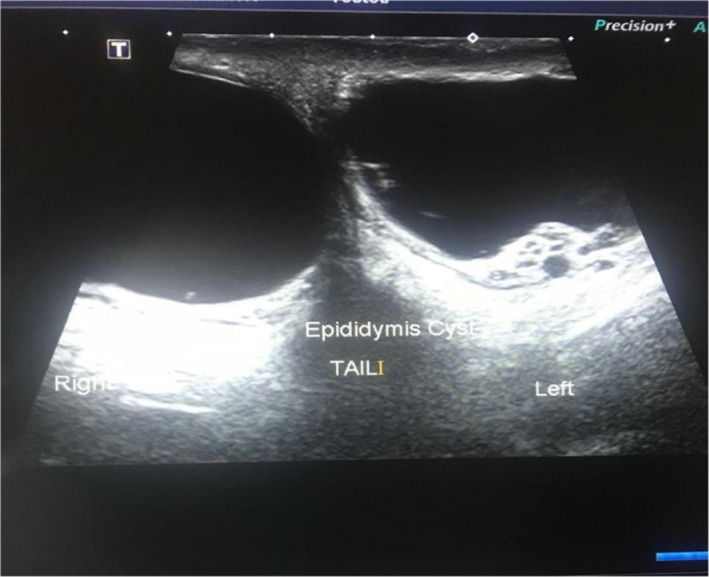
Demonstrating bilateral epididymal cysts arising from the tail of each epididymis

A 16‐year‐old male presented with a 12‐month history of progressively enlarging bilateral painless scrotal lumps with no scrotal size variation throughout the day and no history of trauma. The physical examination revealed normal testes in size and location and bilateral nonreducible nontender oval soft scrotal masses palpated adjacent to both testes (Figure 1). The clinical differential diagnoses included bilateral epididymal cysts, bilateral hydroceles of the spermatic cord, bilateral inguinoscrotal hernias. Scrotal ultrasound outlined two large cystic masses in both epididymal tails measuring 46 × 21 mm on the left and 46 × 24 mm on the right (Figure 2). Both testes appeared normal in size, shape, and echogenicity with normal color flow Doppler appearances. Testicular tumor markers including lactate dehydrogenase (LDH), human chorionic gonadotropin (HCG), and alpha‐fetoprotein (AFP) were within normal ranges. The patient and his father were counselled on conservative management but insisted on cyst excision. Therefore, he was put on our elective waiting list and a 3‐month follow‐up date given. They presented 6 weeks later before any of their appointments to report spontaneous resolution of the cyst on the right (Figure 1B) which was confirmed on scrotal ultrasound and again 2 weeks later to report resolution of cyst on the left which was confirmed clinically.

## 3 DISCUSSION

Epididymal cysts are unilocular or multilocular collection of fluid in the epididymis as a result of dilatation of efferent epididymal tubules due to tubular obstruction.^1^, ^2^ A study found that local production of proinflammatory cytokines evidenced by elevated concentrations of interleukin 8 (IL‐8), interleukin 6 (IL‐6) in epididymal cyst is responsible for cyst formation.^4^ They usually develop in adult men and mostly unilateral. Although uncommon in pediatric patients, they can be found in a number of boys in puberty. Bilateral epididymal cysts are less frequent.^5^ In our case, the patient was 16 years old and had bilateral epididymal cysts which occur less commonly in this age group.

However, they can be symptomatic or not with different clinical presentations such as scrotal swelling which may be narrated by the patient as third testicle; they can present with testicular pain or orchalgia; they can be incidentally found during physical examination or detected on scrotal ultrasound examination. Clinically, epididymal cysts are palpated as extratesticular masses usually smooth, round, and characteristically located within the epididymis.^6^ These epididymal cysts are usually translucent as they contain clear fluid but few due to the presence of spermatozoa may have turbid fluid.^1^ Our patient did not have any pains at all, scrotal swelling was the only bothersome complaint that lead to ultrasound examination confirming the diagnosis.

Ultrasound examination is helpful in locating the cyst within the epididymis. The most common site of origin is the head of epididymis but can rarely arise from body and tail.^1^, ^7^ Ultrasonographic appearance of epididymal cyst is similar to those of cystic tumors such as adenomatoid tumor of epididymis, epidermoid cyst (monodermal teratoma) of the testis. They should also be considered as differential diagnosis.^5^ Epididymal cyst has also been associated with conditions like polycystic kidney disease, cystic fibrosis, Von Hippel‐lindau disease and also reported in children exposed to diethylstilbestrol in utero life.^1^

Treatment for epididymal cysts includes conservative management, aspiration of cyst, sclerotherapy, and excision of cyst.^1^ Conservative management has been suggested in most cases although there is no consensus about the most adequate therapy of pubertal patients and the majority of these cysts involute with time.^5^ The average time to complete involution varies from 4 to 50 months.^2^ A study by BK Arora et al, reported that no spontaneous resolution was observed in the group of patients with cysts between 21 and 50 mm over a 1‐year follow‐up period.^1^ Nonetheless, our patient cyst sizes were within this group ranges and involution occurred just over 1 year from the onset. The patient and parents were counselled about possible spontaneous resolution which can take up to 4 years before it occurs but were insistent in having the surgery planned. Fortunately, complete involution occurred in about 14 months from onset while waiting for an elective surgical date. However, if they become symptomatic, suddenly start increasing in size, or do not resolve spontaneously, nonconservative management should be considered such as surgery or sclerotherapy. However, Niedzielski et al, do not approve and recommend sclerotherapy in the developing testes of pubertal boys.^5^

## 4 CONCLUSION

Conservative management with spontaneous resolution may still occur with no need for surgical excision of cysts if they are asymptomatic even when increasing in size of the cysts may be the only indication for surgical management.

## CONFLICT OF INTEREST

None declared.

## AUTHOR CONTRIBUTIONS

AMM: Substantial contributions to conception and design of the case report; Acquisition of data; drafting of the manuscript; critical revision for important intellectual content; and approval of final version.

## CONSENT

Written informed consent was obtained from the patient for publication of this manuscript and accompanying pictures. A copy of the written consent is available for review by the Editor‐in‐Chief of this journal.
